# Supplementary material for: A novel approach to immunoapheresis of C3a/C3 and proteomic identification of associates
Source: PeerJ. 2019 Dec 16;7:e8218. doi: 10.7717/peerj.8218 (PMC6921979; doi:10.7717/peerj.8218)
Supplement: Supplemental Information 1 — Further potential C3-interacting partners, with proteins below selected cutoff criteria and enrichment value above 0, located in the right half of the volcano plot of Fig. 5. [file peerj-07-8218-s001.docx]

| **Protein Name** | **Log2 C3 Enrichment** | **Log2 (p-value)** |
| --- | --- | --- |
| Plasma protease C1 inhibitor | 3.389 | -4.169 |
| Complement C4-A | 3.323 | -3.699 |
| Desmocollin-3 | 3.280 | -7.192 |
| Basigin | 3.247 | -2.334 |
| Heat shock protein HSP 90-beta | 3.170 | -1.546 |
| Epiplakin | 3.136 | -5.553 |
| Filamin-B | 3.120 | -5.472 |
| Cystatin-M | 3.048 | -3.030 |
| Small nuclear ribonucleoprotein Sm D2 | 3.006 | -1.263 |
| Protein ERGIC-53 | 2.977 | -1.594 |
| 60S ribosomal protein L4 | 2.938 | -1.266 |
| Suprabasin | 2.925 | -5.053 |
| Vitronectin | 2.889 | -6.861 |
| ATP synthase subunit alpha, mitochondrial | 2.872 | -2.243 |
| 40S ribosomal protein S10 | 2.793 | -1.274 |
| Cofilin-1 | 2.790 | -2.392 |
| Periplakin | 2.779 | -5.477 |
| Nicotinamide phosphoribosyltransferase | 2.739 | -1.583 |
| Mannan-binding lectin serine protease 1 | 2.725 | -2.894 |
| Heterogeneous nuclear ribonucleoproteins A2/B1 | 2.709 | -2.374 |
| Heterogeneous nuclear ribonucleoprotein A1 | 2.675 | -1.441 |
| Alpha-2-antiplasmin | 2.670 | -2.647 |
| Kynureninase | 2.637 | -2.020 |
| IgGFc-binding protein | 2.563 | -8.545 |
| Complement factor H-related protein 5 | 2.560 | -2.153 |
| Fibulin-1 | 2.559 | -4.251 |
| Hepatoma-derived growth factor | 2.548 | -1.581 |
| Calumenin | 2.457 | -2.688 |
| Glucosidase 2 subunit beta | 2.433 | -1.310 |
| Bleomycin hydrolase | 2.422 | -4.859 |
| Ras GTPase-activating protein-binding protein 1 | 2.419 | -2.795 |
| Nucleolin | 2.409 | -3.306 |
| N-acetylmuramoyl-L-alanine amidase | 2.351 | -9.014 |
| Kallikrein-7 | 2.350 | -4.882 |
| Nucleophosmin | 2.315 | -2.361 |
| Arginase-1 | 2.304 | -10.733 |
| Desmocollin-1 | 2.242 | -5.937 |
| Syntaxin-binding protein 5 | 2.217 | -3.115 |
| Pregnancy zone protein | 2.208 | -4.767 |
| Neutral alpha-glucosidase AB | 2.174 | -1.467 |
| Keratinocyte proline-rich protein | 2.155 | -13.263 |
| 14-3-3 protein zeta/delta | 2.154 | -1.937 |
| Histone-binding protein RBBP7 | 2.095 | -1.383 |
| Angiotensinogen | 2.066 | -1.441 |
| Serum amyloid P-component | 2.063 | -8.568 |
| Desmoglein-1 | 2.053 | -5.730 |
| Stress-70 protein, mitochondrial | 2.025 | -2.837 |
| Histone H4 | 1.998 | -10.442 |
| Dermcidin | 1.972 | -6.267 |
| Phospholipase B-like 1 | 1.966 | -2.367 |
| Splicing factor, proline- and glutamine-rich | 1.950 | -1.637 |
| Reticulocalbin-1 | 1.922 | -1.450 |
| Protein disulfide-isomerase A4 | 1.887 | -2.476 |
| Plasminogen | 1.862 | -2.357 |
| Plectin | 1.802 | -4.952 |
| Complement C1s subcomponent | 1.786 | -1.697 |
| Heat shock 70 kDa protein 1A | 1.756 | -2.053 |
| ATP synthase subunit beta, mitochondrial | 1.754 | -1.549 |
| 3-hydroxyacyl-CoA dehydrogenase type-2 | 1.742 | -1.558 |
| Keratin, type I cytoskeletal 18 | 1.730 | -1.567 |
| Vimentin | 1.725 | -1.773 |
| Serpin H1 | 1.687 | -1.696 |
| Interleukin enhancer-binding factor 2 | 1.668 | -1.620 |
| Inosine-5'-monophosphate dehydrogenase 2 | 1.657 | -1.968 |
| Clathrin heavy chain 1 | 1.638 | -1.795 |
| Envoplakin | 1.622 | -4.278 |
| 60S ribosomal protein L18a | 1.612 | -4.401 |
| Protein disulfide-isomerase A3 | 1.609 | -1.678 |
| Moesin | 1.608 | -1.679 |
| 4F2 cell-surface antigen heavy chain | 1.583 | -1.707 |
| Keratin, type II cytoskeletal 72 | 1.567 | -4.437 |
| Procollagen-lysine,2-oxoglutarate 5-dioxygenase 1 | 1.470 | -1.861 |
| Endoplasmic reticulum chaperone BiP | 1.464 | -2.115 |
| Acid ceramidase | 1.406 | -2.525 |
| Calnexin | 1.389 | -1.866 |
| 14-3-3 protein beta/alpha | 1.368 | -2.054 |
| RuvB-like 2 | 1.354 | -2.086 |
| Ubiquitin-like modifier-activating enzyme 1 | 1.353 | -1.637 |
| Serine hydroxymethyltransferase, mitochondrial | 1.339 | -2.120 |
| Serpin B12 | 1.261 | -6.443 |
| Tubulin beta-3 chain | 1.211 | -1.832 |
| Complement C5 | 1.138 | -2.791 |
| Inter-alpha-trypsin inhibitor heavy chain H3 | 1.129 | -2.874 |
| tRNA-splicing ligase RtcB homolog | 1.106 | -2.938 |
| Transferrin receptor protein 1 | 1.098 | -2.569 |
| RuvB-like 1 | 1.070 | -3.116 |
| HLA class I histocompatibility antigen, Cw-3 alpha chain | 1.041 | -5.545 |
| Importin subunit beta-1 | 1.037 | -3.280 |
| Apolipoprotein A-IV | 1.022 | -0.648 |
| Neuroblast differentiation-associated protein AHNAK | 0.999 | -2.338 |
| Glucosylceramidase | 0.982 | -4.365 |
| Peroxiredoxin-4 | 0.947 | -3.671 |
| Peroxisomal multifunctional enzyme type 2 | 0.942 | -3.121 |
| Alpha-actinin-4 | 0.880 | -2.072 |
| Protein-glutamine gamma-glutamyltransferase K | 0.878 | -7.604 |
| AP-1 complex subunit beta-1 | 0.871 | -3.717 |
| Alpha-enolase | 0.859 | -1.210 |
| Eukaryotic translation initiation factor 4 gamma 1 | 0.833 | -3.589 |
| DNA-dependent protein kinase catalytic subunit | 0.817 | -3.509 |
| Deoxyuridine 5'-triphosphate nucleotidohydrolase, mitochondrial | 0.798 | -3.403 |
| Extracellular matrix protein 1 | 0.759 | -3.728 |
| Peroxiredoxin-6 | 0.731 | -2.936 |
| Transitional endoplasmic reticulum ATPase | 0.726 | -2.169 |
| Histidine ammonia-lyase | 0.709 | -2.449 |
| Annexin A11 | 0.675 | -1.734 |
| Apolipoprotein E | 0.575 | -3.028 |
| Filamin-A | 0.572 | -1.937 |
| Cathepsin L2 | 0.553 | -2.134 |
| Filaggrin-2 | 0.500 | -3.332 |
| Complement C1r subcomponent | 0.463 | -2.904 |
| Pyruvate kinase PKM | 0.324 | -1.126 |
| Isocitrate dehydrogenase [NADP] cytoplasmic | 0.314 | -1.540 |
| F-box only protein 50 | 0.285 | -0.911 |
| Plakophilin-1 | 0.282 | -1.387 |
| Tropomyosin beta chain | 0.281 | -0.175 |
| Fibronectin | 0.272 | -1.811 |
| 40S ribosomal protein S3 | 0.234 | -0.139 |
| Vinculin | 0.222 | -0.658 |
| Lysosome-associated membrane glycoprotein 1 | 0.206 | -0.312 |
| NADP-dependent malic enzyme | 0.086 | -0.366 |
| Puromycin-sensitive aminopeptidase | 0.059 | -0.359 |
| Alpha-1-acid glycoprotein 2 | 0.056 | -0.561 |
| Alpha-1-antichymotrypsin | 0.028 | -0.300 |
|  |  |  |
