# Supplementary figures and images for: A novel approach to immunoapheresis of C3a/C3 and proteomic identification of associates
Source: PeerJ. 2019 Dec 16;7:e8218. doi: 10.7717/peerj.8218 (PMC6921979; doi:10.7717/peerj.8218)

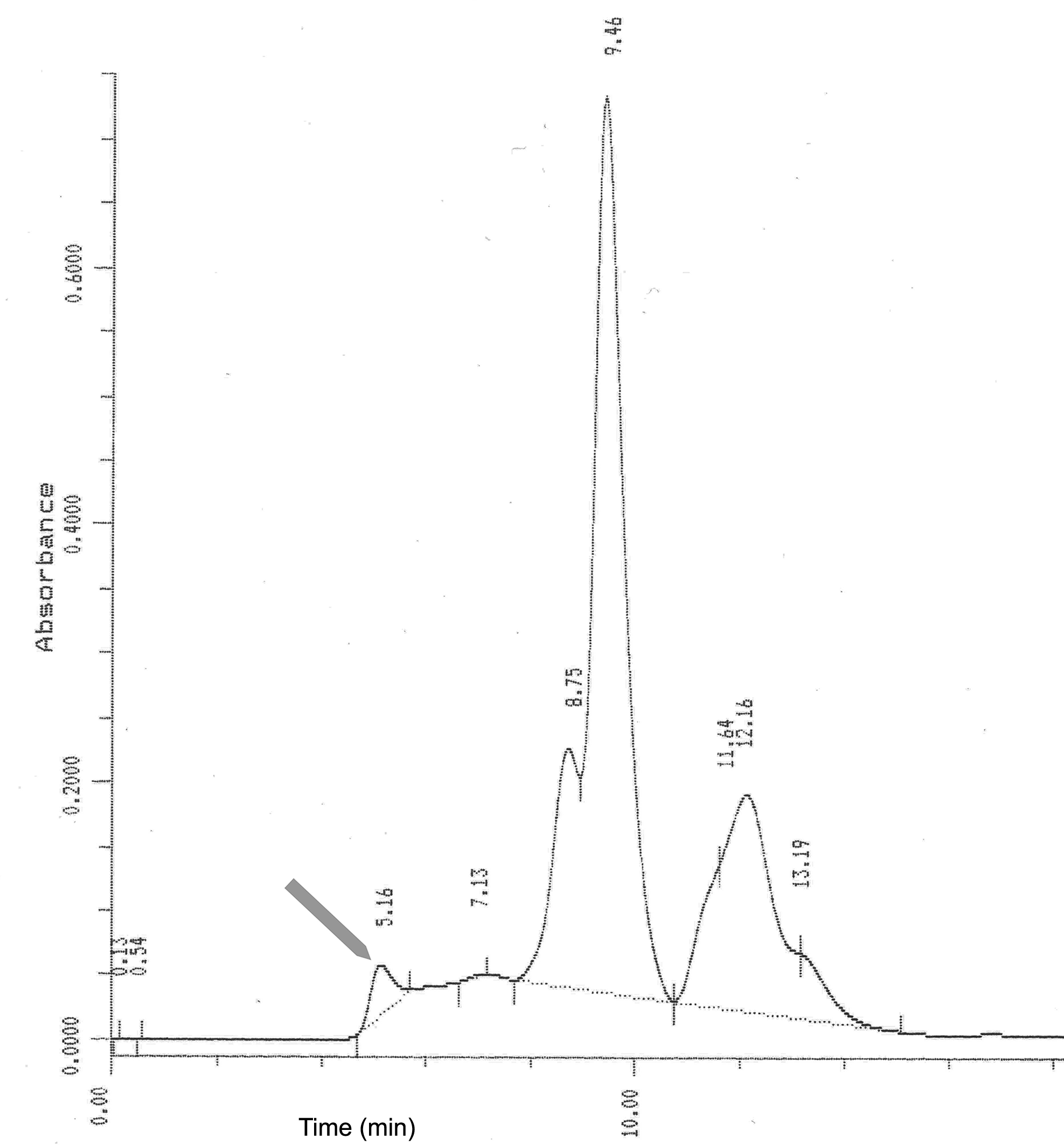

Supplement: Supplemental Information 2 — The gray arrow points at the protein peak eluted at 5.16 min used as immunogen for mouse immunization. A representative chromatogram out of five experiments is presented. [file peerj-07-8218-s002.png]

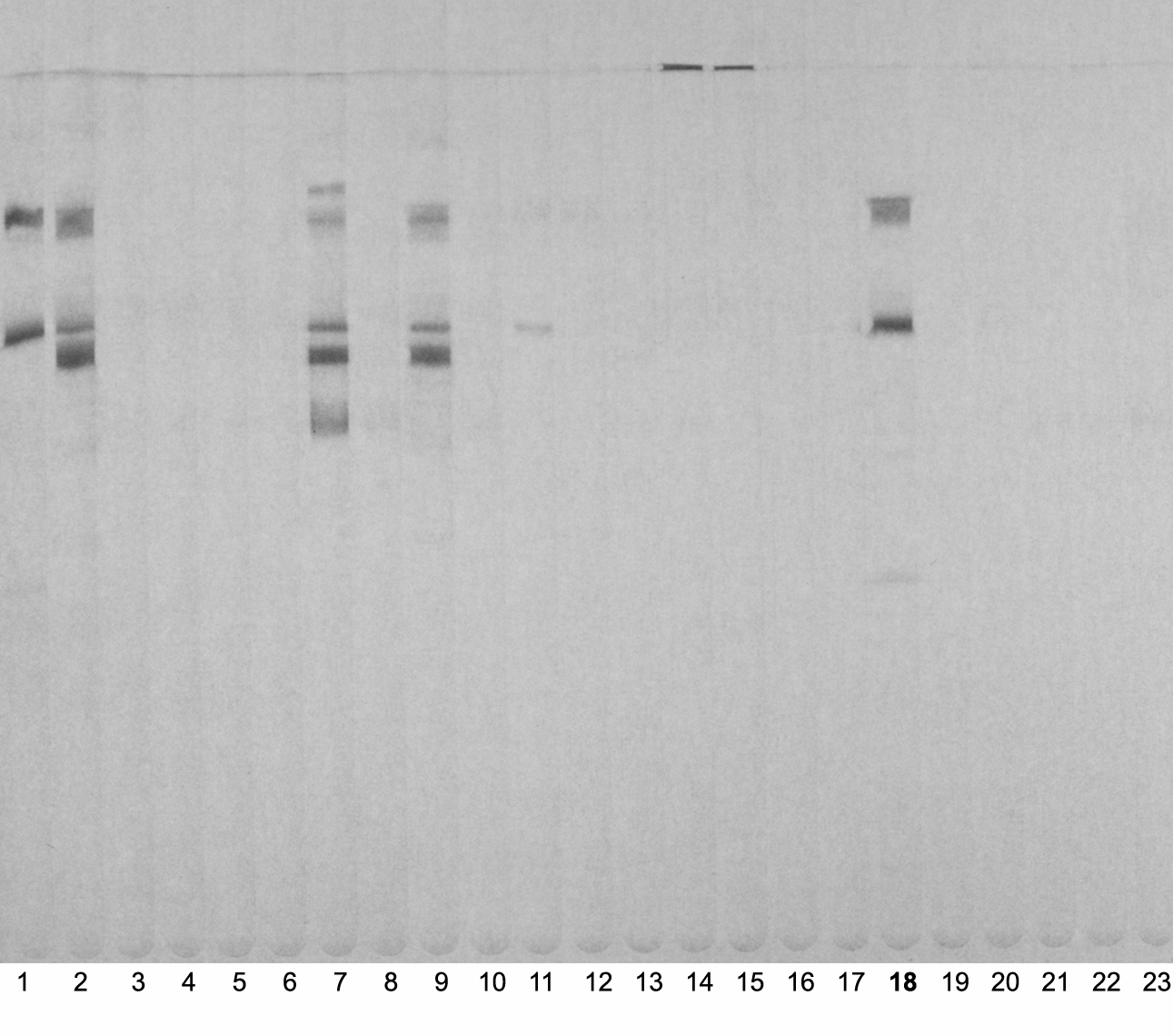

Supplement: Supplemental Information 3 — Two hundred µg of original purified immunogen was loaded onto a flat-comb gel. The blotted membrane was inserted into the membrane processor (Milliblot-MP; Millipore, Bedford, MA, USA) and 250 µL of tissue culture supernatant obtained from individual wells was loaded into each lane. After incubation of 180 min at room temperature under constant shaking followed by two washing steps for 10 min each with TPBS, the primary antibody was followed by a goat anti mouse POX conjugated Ab 1:10,000. After 60 min incubation at room temperature the blot was finally developed using 4 chloronapthol/H2O2 chromogen/substrate mixture. Lane 18 represents clone 3F7E2. [file peerj-07-8218-s003.png]

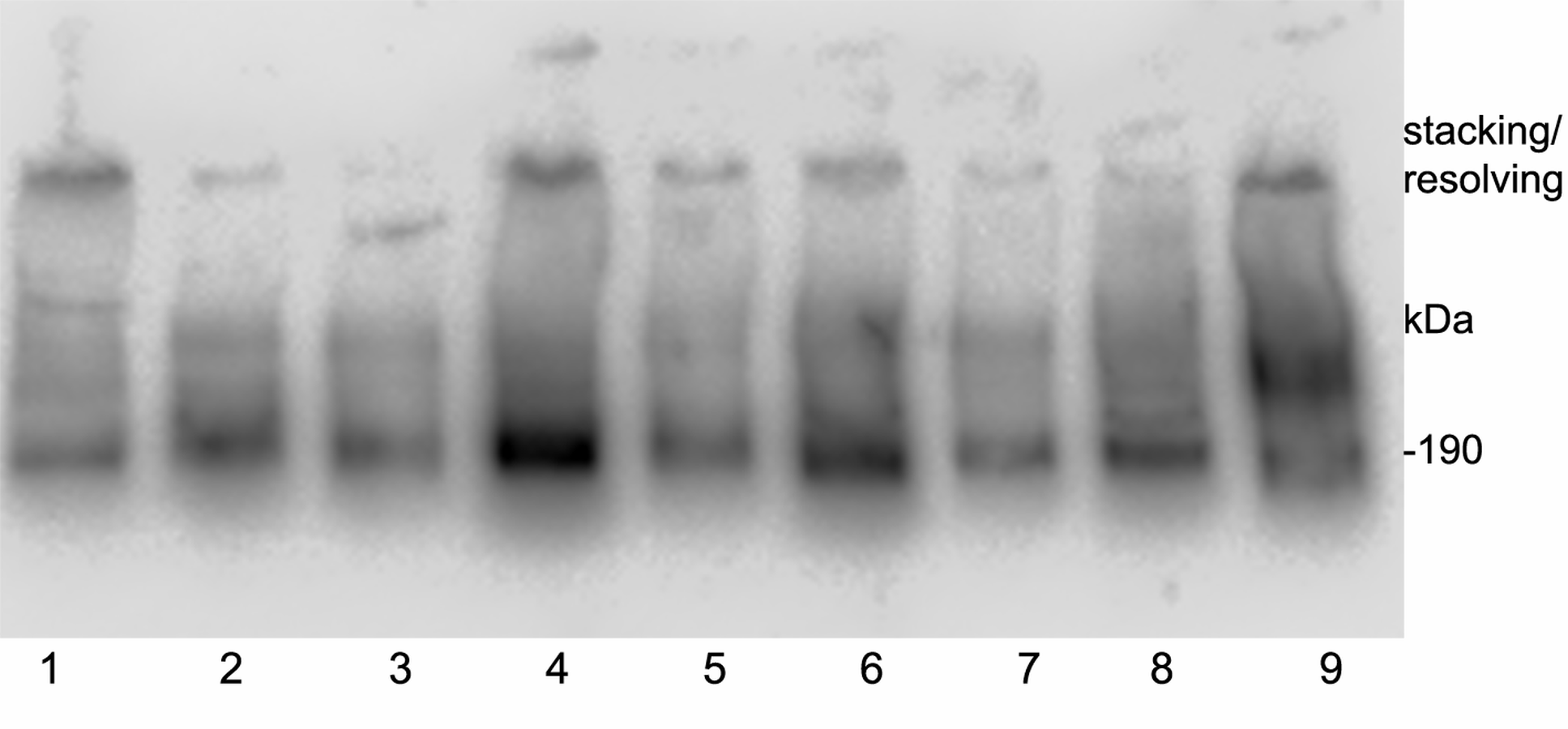

Supplement: Supplemental Information 4 — One µL of human serum was loaded onto each lane of a 10% PAGE gel omitting SDS in sample and running buffer, which was eloctrotransferred onto nitrocellulose. The 3F7E2-antibody binding site was developed using HRP conjugated goat anti mouse Ab and chemoluminescence reagent. The immunoblot revealed a spreading of C3 positive bands over a broad range in each of the nine tested individuals and the pattern was unique to each individual. The main dominant band in all samples was at a size of 190 kDa, which complies with the expected size of native C3. [file peerj-07-8218-s004.png]

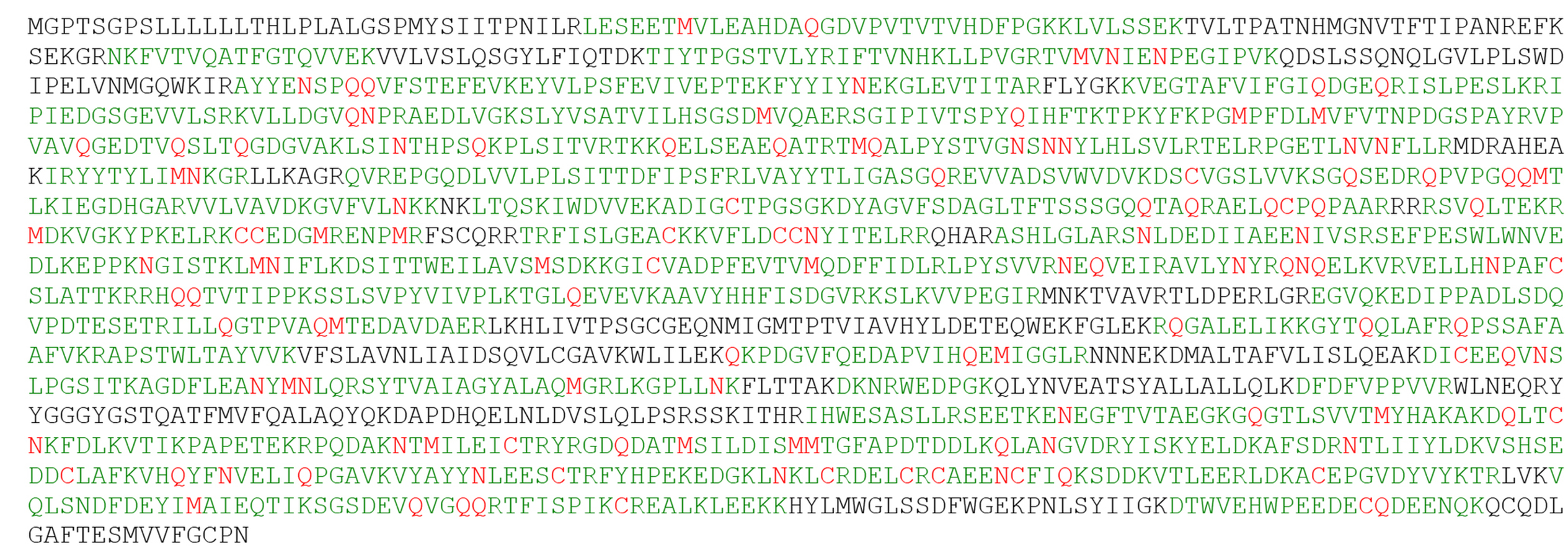

Supplement: Supplemental Information 5 — Peptides identified by mass spectrometry from band at 190 kDa covering the C3 protein sequence are indicated in green color; potential modifications are indicated in red. [file peerj-07-8218-s005.png]
